# Enigmatic presentation of glomus tumour of the tongue: diagnostic insights

**DOI:** 10.3332/ecancer.2025.2006

**Published:** 2025-10-06

**Authors:** Nairica Eurico Rebello, Anita Spadigam, Anita Dhupar, Jochima Cota, Vikas Dhupar

**Affiliations:** 1 Department of Oral and Maxillofacial Pathology, Goa Dental College and Hospital, Bambolim, Goa 403202, India; 2 Department of Oral and Maxillofacial Surgery, Goa Dental College and Hospital, Bambolim, Goa 403202, India

**Keywords:** case report, glomangioma, glomus tumour, oral cavity, TLE1 protein, tongue neoplasm

## Abstract

Glomus tumour is a soft tissue neoplasm that is associated with the subungual region owing to the presence of the glomus body. A 53-year-old female patient was referred to the Department of Oral and Maxillofacial Pathology with the chief complaint of a swelling on the tongue of 3 months' duration. An ultrasound of the tongue suggested a vascular malformation. Histopathology revealed a well-defined proliferation of cells around blood vessels, surrounded by muscle and collagen fibres. A majority of the cells were clear with distinct nuclei. Some cells had an eosinophilic granular cytoplasm. Histopathology was augmented with immunohistochemistry to arrive at a diagnosis of glomus tumour of the tongue. The oral cavity is an unusual site for the occurrence of glomus tumour. This case emphasises the importance of a comprehensive diagnostic work-up in the diagnosis of swellings in the head and neck region, particularly in the context of rare tumours arising from cells that are not native to this site.

## Background

Glomus tumour is an uncommon neoplasm representing 2% of all soft tissue tumours [1]. It seldom presents in the head and neck region and is exceedingly rare in the oral cavity [1]. It is hypothesised to originate from the glomus apparatus, a specialised arteriovenous anastomosis that regulates heat and is located in the stratum reticularis of the dermis [1].

We present the case of an intraoral glomus tumour in a 53-year-old female patient with a painless swelling on the lateral border of the tongue. We discuss the unusual features of this rare entity and the diagnostic work-up we employed to arrive at a definitive diagnosis.

## Case presentation

A 53-year-old female reported to us with the complaint of a swelling on the left side of the tongue of 3 months duration.

The patient gave a history of trauma on mastication, gradual increase in size of swelling and no history of pain or discharge in relation to the swelling. Past medical history included a hysterectomy 10 years prior. Past dental history was unremarkable. The patient consumed a vegetarian diet, had an adequate appetite and did not have any deleterious habits. Family history was unremarkable. General examination revealed a well-built female, who was well oriented in time, place and person. Icterus, pallor and cyanosis were absent. Intraoral examination revealed an ovoid swelling measuring approximately 3 × 2 cm in diameter involving the left lateral border of the tongue. The swelling was sessile and firm with a rough surface and well-defined margins. It was non-fluctuant, non-compressible and did not bleed or blanch on palpation. Regional lymphadenopathy was absent.

**Figure 1.**
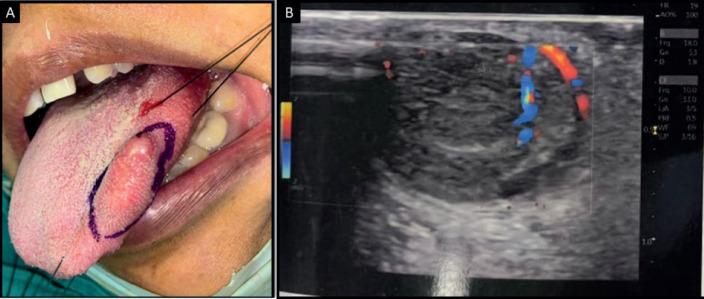
(a): Intraoral photograph showing swelling on the left lateral border of the tongue. (b): Ultrasonography of tongue showing mildly heterogenous lesion with smooth margins and vascularity.

Ultrasonography of the tongue revealed a solid-appearing, mildly heterogenous lesion with smooth margins and vascularity within it, which was suggestive of a vascular malformation. Plain and post-contrast magnetic resonance imaging of the tongue revealed a well-defined ovoid T2-weighted hyperintense lesion, which appeared isointense on T1-weighted imaging and was suggestive of a cystic lymphangioma of the tongue (Figure 1). No abnormality was detected on computed tomography of the chest and abdominal ultrasound.

**Figure 2.**
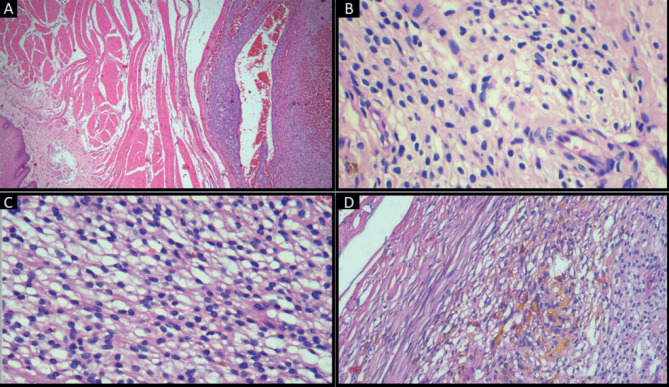
Haematoxylin and eosin-stained section of tumour showing: (a): Tumour cells surrounded by smooth muscle fibres (original magnification ×4); (b): Clear cells with punched out nuclei (original magnification ×40); (c): Clear cells (original magnification ×40); (d): Hematoidin pigment (original magnification ×40).

Holding sutures were placed on the tip of the tongue and the postero-lateral aspect for ease of manipulation. Local anaesthesia (lignocaine 2% with 1:200000 adrenaline was injected and the swelling was excised. Bleeding was stopped with cauterisation and sutures were placed. The sample was sent for histopathological evaluation. Histopathology revealed stratified squamous parakeratinised epithelium overlying a fibro-cellular connective tissue stroma. The superficial stroma consisted of a well-circumscribed proliferation of predominantly round to ovoid cells in a perivascular arrangement that were surrounded by muscle fibres at the periphery. The cells had well-defined cell margins, clear to pale eosinophilic cytoplasm and vesicular nuclei. Focal areas showed spindle cells and ovoid cells with granular cytoplasm. Blood vessels of varying calibres and large areas of haemorrhage were present. This finding was consistent with the vascularity noted on the ultrasonogram and the isointense areas noted on the magnetic resonance imaging. Perivascular hyalinisation was noted, with some blood vessels showing a staghorn morphology. Patchy areas of hematoidin pigment were noted (Figure 2).

**Figure 3.**
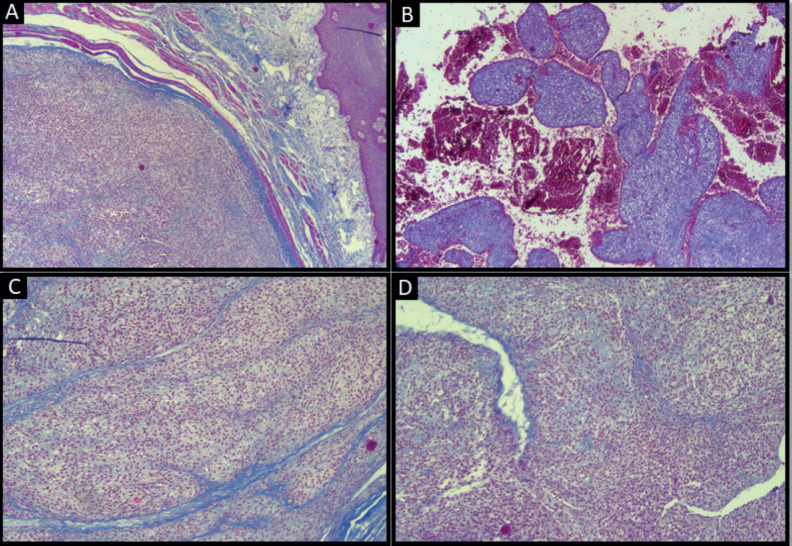
Masson’s trichrome-stained section of tumour showing: (a): Smooth muscle fibres (original magnification ×4); (b): Haemorrhage (original magnification ×4); (c): Collagen fibre septae (original magnification ×10); (d): Staghorn-shaped vessels (original magnification ×10).

Atypia and necrosis were absent. Masson’s trichrome-stained sections highlighted the intramuscular location of the tumour, organoid arrangement of tumour cells separated by septae of collagen fibres, large areas of haemorrhage and staghorn morphology of blood vessels (Figure 3).

**Figure 4.**
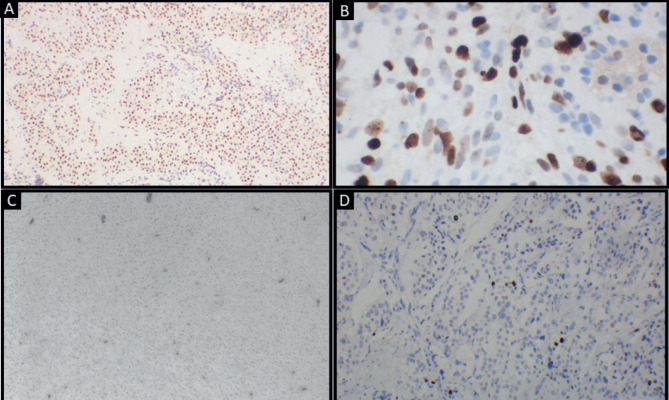
Tumour cells showing: (a): Diffuse positivity for alpha smooth muscle actin (original magnification ×4); (b): Diffuse positivity for TLE1 (original magnification ×40); (c): Focal positivity for CD34 (original magnification ×10); (d): Clusters of cells showing high proliferative activity (original magnification ×10). (Immunohistochemistry).

Differential diagnosis included solitary fibrous tumour, myopericytoma, PEComa, glomus tumour and synovial sarcoma. Immunohistochemistry revealed diffuse positivity for alpha smooth muscle actin and TLE1 and focal CD34 positivity. Cells were negative for p63, HMB45, Melan A, SS18 and S100. Focal areas showed ki67 proliferative index of 20% (Figure 4).

The above features were consistent with the diagnosis of glomus tumour with high proliferative potential. The patient has been advised follow-up every 3 months and is currently disease free.

## Discussion

Glomus tumours of the head and neck region account for less than 1% of all glomus tumours [2]. They typically present as red or blue subcutaneous nodules, characterised by a triad of symptoms: hypersensitivity to cold, stabbing pinpoint pain and paroxysmal pain [3]. The intraoral counterparts, in contrast, are associated with mild pain [3]. Head and neck glomus tumours are larger than their extraoral counterparts, measuring approximately 1–1.5 cm [2]. Most cases have been reported on the lips followed by the hard palate, tongue, buccal mucosa and gingiva [2]. Table 1 lists all cases of intraoral glomus tumours diagnosed with histopathology in conjunction with immunohistochemistry [12–28]. The recurrence rate of glomus tumours in the head and neck region is yet to be quantified. However, the likelihood of recurrence is higher in cases of incomplete resection of malignant glomus tumours [1]. Ultrasonography, though non-specific, plays a vital role in early diagnosis, offers precise locational details and assists in guiding tumour excision [4]. The present case warranted the use of magnetic resonance imaging to augment the findings obtained on ultrasonography. The findings were consistent with those of del Carpio *et al* [4] who characterised glomus tumours as T2-weighted hyperintense lesions owing to high vascularity.

The first description of this entity as described by Masson highlighted three distinct histological patterns that usually exist in the same case: the angiomatous zone, characterised by large blood vessels (most common); the solid zone, comprising clusters of epithelioid and smooth muscle cells; and the degenerating zone, marked by hyaline or myxoid changes [5]. The World Health Organisation proposed a categorisation based on the predominant cell population: solid, predominantly cellular with very few vascular channels; glomangioma, an abundance of blood vessels with an admixture of cells surrounding the vessels; and glomangiomyoma, cells resembling smooth muscle are abundant with an admixture of vascular channels and glomus cells [1,3]. The solid pattern has been reported as the most common in intraoral tumours, and more than one histological pattern may exist in the same tumour [5]. The present case revealed a combination of solid and glomangioma-like areas. Areas with signet ring cells and oncocyte-like granular cells similar to those reported in the literature [1] were also noted (Figure 2c). The closest histopathological mimic in the present case was PEComa. Perivascular Epithelioid Cell tumours are characterised by nests and sheets of epithelioid cells located in close proximity to the walls of blood vessel. These tumours exhibit dual differentiation, expressing both smooth muscle and melanocytic markers, reflecting their myo-melanocytic origin [6]. A lack of expression of melanocytic markers (Melan A, HMB45) validated exclusion of this entity form our differential diagnosis.

There exists a limited number of cases showing CD34 positivity in tumour cells [7]. The present case exhibited focal areas of CD34 positivity. Bozdogan *et al* [8] evaluated TLE-1 expression in 22 cases of glomus tumour following an incidental finding and reported that 91.6% of cases showed TLE-1 positivity. Subsequently, no studies have evaluated the TLE-1 status of glomus tumours. The current case showed diffuse TLE-1 positivity in all tumour cells.

Atypical variants were classified as follows by Folpe *et al* [9]: i) malignant glomus tumour, ii) symplastic glomus tumour, which is identified as glomus tumour with nuclear atypia only, (iii) glomus tumour of uncertain malignant potential and iv) glomangiomatosis (histologically benign glomus tumour exhibiting diffuse growth).

Despite the rarity of this neoplasm in the head and neck region, a few cases of glomangiosarcoma have been reported, including a case with intracranial metastasis and a case of glomangiosarcoma from a distant site with metastasis to the jaw [10].

Owing to the benign nature of this neoplasm, a ki67 index of up to 5% has been reported in most cases [2, 5, 10]. Rajendran *et al* [11] reported a case of glomangiosarcoma with ki67 index of 15%–20% with features of mild atypia and <5 mitoses/50 HPF. The present case demonstrated a ki67 index of 20%, which is unusual. However, a lack of atypia and mitosis warranted a diagnosis of benign glomus tumour.

The recurrence rate of glomus tumours in the head and neck region is yet to be quantified. However, the likelihood of recurrence is higher in cases of incomplete resection of malignant glomus tumours [1].

## Conclusion

The present case highlights the difference in the clinical presentation of intraoral glomus tumours in comparison with those in extraoral sites. These differences complicate diagnosis and may contribute to an underestimation of the prevalence of this entity. An accurately formulated diagnostic algorithm is essential to prevent misdiagnosis and initiate treatment and follow-up.

## Conflicts of interest

No conflicts to disclose.

## Funding

No funding was received.

## Informed consent

Written consent for publication was obtained from the patient.

## Author contributions

NR, AS, AD and JC: Preparation, creation and presentation of the paper, drafting of the manuscript and supervision. NR, AS and VD: Provision of anatomic pathology materials. All authors approved the manuscript for submission.

## Figures and Tables

**Table 1.** Intraoral glomus tumours diagnosed with histopathology in conjunction with immunohistochemistry.

Age	Gender	Site	IHC		Treatment	Reference
			Positive	Negative		
31	M	Lower lip	SMA, MSA, S100	CK, EMA, CD34, CD31, chromogranin	Surgical excision	[12]
11	F	Lower lip	SMA, Vimentin	Factor XIII	Surgical excision	[13]
71	M	Palate	-	Actin, Desmin, Chromogranin, NSE, PGP9.5	Surgical excision	[14]
54	M	Upper lip	SMA, MSA	-	Surgical excision	[15]
57	M	Upper lip	SMA, MSA	-	Surgical excision	[15]
46	F	Palate	Vimentin, S100	Desmin, Actin, Chromogranin, NSE, Cytokeratin, EMA, Factor VIII	Surgical excision	[16]
57	M	Upper lip	Actin, Desmin, Vimentin, S100	-	Surgical excision	[17]
65	F	Upper lip	Vimentin	CD45, Factor VIII, Cytokeratin	Surgical excision	[18]
54	M	Upper lip	SMA, Vimentin	Factor VIII	Surgical excision	[19]
45	M	Left buccal mucosa	Actin, SMA		Surgical excision	[20]
67	F	Upper lip	SMA, CD34	Vimentin, S100, Cytokeratin	Surgical excision	[21]
30	F	Buccal mucosa	SMA, Vimentin	CD31, CD34, Desmin, S100, Cytokeratin, CD117, LCA, BCL2	Surgical excision	[22]
58	F	Ventrum of tongue	SMA, MSA	P63, S100, GFAP, Chromogranin, Sox10, AE1/3, CD31, CD34	Surgical excision	[1]
26	M	Lower lip	SMA, MSA	STAT6, S100, CD31, CD34, AE1/3	Surgical excision	[1]
37	M	Buccal mucosa	Vimentin, SMA, CD34	AE1/3, CD31, S100	Laser excision	[5]
8	F	Mandibular alveolus	Vimentin, SMA	Desmin, CD34, p63, CD45	Surgical excision	[23]
46	M	Upper lip	Vimentin, SMA, CD34	S100, Desmin, CD117, Pan-cytokeratin, p63, β catenin	Surgical excision	[24]
24	F	Floor of the mouth	Vimentin, SMA	Desmin, CD31, CD34, AE 1/3	Surgical excision	[25]
62	M	Lower lip	SMA	-	Surgical excision	[10]
51	F	Upper lip	Vimentin, CD34, SMA, HHF-35, h-Caldesmon	AE1/AE3, CD138, S-100 Desmin	Surgical excision	[26]
44	M	Mandible	Vimentin, MSA/HHF35, Calponin	SMA, AE1/AE3, Cytokeratin (CAM5.2), CK19, CD31, CD34, CD68, p63, S-100, Factor VIII, Desmin	Surgical excision	[27]
21	M	Palate	Vimentin, SMA, HHF-35	Keratin, Desmin, S-100, factor VIII	Surgical excision	[28]
